# Ramulus Mori (Sangzhi) alkaloids ameliorate high-fat diet induced obesity in rats by modulating gut microbiota and bile acid metabolism

**DOI:** 10.3389/fendo.2024.1506430

**Published:** 2024-12-20

**Authors:** Xin Shang, Yu Fu, Ying Wang, Shuxun Yan

**Affiliations:** ^1^ Department of Endocrinology, The First Affiliated Hospital of Henan University of Chinese Medicine, Zhengzhou, China; ^2^ School of First Clinical, Henan University of Chinese Medicine, Zhengzhou, China; ^3^ Department of Geriatrics, First Affiliated Hospital of Zhengzhou University, Zhengzhou, China

**Keywords:** obesity, Ramulus Mori (Sangzhi) alkaloids, gut microbiota, lipid metabolism, BA metabolism

## Abstract

**Objective:**

The objective of this study is to investigate the ability of Ramulus Mori (Sangzhi) alkaloid tablets (SZ-A) to ameliorate obesity and lipid metabolism disorders in rats subjected to a high-fat diet (HFD) through metagenomics, untargeted lipidomics, targeted metabolism of bile acid (BA), and BA pathways, providing a novel perspective on the management of metabolic disorders.

**Methods:**

In this research, HFD-fed rats were concurrently administered SZ-A orally. We measured changes in body weight (BW), blood lipid profiles, and liver function to assess therapeutic effects. Liver lipid status was visualized through H&E and Oil Red O. Gut microbiota composition was elucidated using metagenomics. The LC-MS-targeted metabolomics approach was utilized to define the fecal BA profiles. Furthermore, the lipid metabolomics of adipose tissue samples was investigated using an LC-MS analysis platform. The expression levels of the BA receptor were determined by western blotting. Additionally, serum insulin (INS), glucagon-like peptide-1 (GLP-1), and inflammatory cytokines were quantified using an ELISA kit. The integrity of the colonic epithelial barrier was assessed using immunofluorescence.

**Results:**

SZ-A notably decreased BW and blood lipid levels in obese rats while also alleviating liver injury. Additionally, SZ-A reduced the serum levels of leptin (LEP), INS, and GLP-1, indicating its potential to modulate key metabolic hormones. Most notably, SZ-A substantially improved gut microbiota composition. Specifically, it reshaped the gut microbiota structure in HFD-fed rats by increasing the relative abundance of beneficial bacteria, such as *Bacteroides*, while decreasing the populations of potentially harmful bacteria, such as *Dorea* and *Blautia*. At the BA level, SZ-A decreased the levels of harmful BAs, including hyodeoxycholic acid (HDCA), deoxycholic acid (DCA), 12-keto lithocholic acid (12-KLCA), lithocholic acid (LCA), and muricholic acid (MDCA). Between the model group and SZ-A, 258 differentially abundant metabolites were detected, with 72 upregulated and 186 downregulated. Furthermore, these BAs are implicated in the activation of the FXR-FGF15 and TGR5-GLP-1 pathways in the intestine. This activation helps to alleviate HFD-fed intestinal inflammation and restore intestinal barrier damage by modulating inflammatory cytokines and bolstering the intestinal barrier’s capabilities.

**Conclusions:**

Our findings indicate that SZ-A effectively modulates BW, serum lipid profiles, and liver function in HFD-fed rats. Moreover, SZ-A exerts a positive influence on inflammatory cytokines, thereby mitigating inflammation and promoting the restoration of the intestinal barrier. Significantly, our research indicates that adjusting the gut microbiome and BA levels could serve as an effective approach for both preventing and treating obesity and related metabolic dyslipidemia.

## 1 Introduction

The growing rates of overweight and obesity have emerged as a pressing global public health challenge. In 2022, the scale of this issue became starkly apparent, with over 890 million adults worldwide experiencing obesity (1). Obesity has become a prevalent condition across all age groups, transcending geographical and demographic boundaries. The alarming surge in global obesity rates has not only affected public health but has also imposed significant economic burdens on most countries (2). With the surge in overweight and obesity, there has been a concurrent transformation in the social and economic landscapes. This shift has been accompanied by significant alterations in lifestyle habits, including dietary patterns and daily routines. The interplay between lifestyle changes and socioeconomic development is exerting a profound and rapid influence on overweight and obese people. The core of being overweight is a mismatch between the number of calories consumed and the energy used, resulting in an overabundance of body fat. This condition is characterized by a complex interplay of factors, with genetics playing a foundational role, yet it is heavily shaped by environmental, dietary, and behavioral factors. Obesity is not merely a cosmetic concern but also a multifactorial disease with profound implications for health. Excess weight and obesity are recognized as contributing factors to numerous long-term health conditions. They are closely linked to type 2 diabetes, heart disease, ongoing mild inflammation, and certain cancers, underscoring the need to tackle the obesity crisis as a key element of public health strategies (3, 4). Nevertheless, existing treatment options for obesity are limited, with certain pharmacological approaches beset by a plethora of conflicting data. The adverse effects of these drugs further complicate the achievement of sustainable weight loss outcomes. Consequently, investigating the pathophysiological mechanisms underpinning obesity and devising efficacious preventative and therapeutic strategies remain critical areas of scientific inquiry.

In recent years, research into the gut microbial community and the processing of BAs has helped in understanding the development of obesity and issues with fat metabolism, as well as in seeking potential treatments (5–7). An increasing body of evidence implicates alterations in the gut microbiota as a significant factor in the progression of obesity (8). Typically, the human gastrointestinal tract is home to a varied and well-balanced microbiome. However, chronic poor dietary habits, medication misuse, and illness can disrupt this delicate equilibrium, leading to dysbiosis. The resulting decline in the intestinal barrier’s strength can trigger endotoxemia, which is associated with widespread inflammation, resistance to insulin, and the onset of metabolic issues like diabetes and the buildup of fats. These factors, in turn, exacerbate the progression of obesity and associated comorbidities (9). BAs are intricately linked to obesity and are subject to regulation by the gut microbiota, serving as crucial metabolic by-products of these microbial communities. Their primary function lies in the absorption, digestion, and emulsification of fats, which are essential for sustaining metabolic equilibrium (10). Predominantly circulating within the hepatic and enteric systems, BAs exert a significant influence on lipid metabolism and are pivotal signaling molecules that modulate metabolic pathways (11). BAs are pivotal in modulating glucose, lipid, and energy metabolism, primarily through the activation of two key receptors: FXR and TGR5 (10, 12). As such, the targeted adjustment of the gut’s microbial environment and BA equilibrium presents itself as a hopeful strategy for managing obesity.

Ramulus Mori (Sangzhi) alkaloids (SZ-A) are bioactive constituents extracted and purified from the traditional Chinese medicinal ingredient ‘Sangzhi’. Distinguished from single-target pharmaceuticals, SZ-A is composed of a rich array of effective compounds, including, but not limited to, flavonoids, polysaccharides, and amino acids. This complex formulation encapsulates the diverse pharmacological benefits inherent in natural medicines. Beyond its well-documented ability to reduce postprandial blood sugar levels, SZ-A is also known to enhance glucose and lipid metabolism, facilitate weight reduction, invigorate the intestinal–pancreatic axis, modulate gut microbiota ecology, and mitigate systemic inflammation, demonstrating its multifaceted therapeutic potential (13–15). Empirical research has demonstrated that SZ-A exerts a pronounced weight-reducing effect in mice and ameliorates conditions associated with non-alcoholic fatty liver disease (NAFLD), inflammation, and fibrosis. Significantly, the 400 mg/kg dosage of SZ-A produced the strongest suppressive impact (16). As a natural medicinal agent, SZ-A possesses the intrinsic potential to modulate metabolic dysregulation and elicit a spectrum of pharmacological responses. This diverse initiative establishes them as potential leaders in addressing metabolic conditions.

In our research, we investigated how SZ-A could treat obesity-linked lipid metabolism issues by adjusting gut bacteria and BA processing. We assessed the safeguarding effects of SZ-A in HFD-fed rats through comprehensive evaluations, including body weight (BW), blood lipid profiles, liver function indices, and histological assessments. Additionally, we utilized metagenomic sequencing and conducted metabolomic analyses of fecal and adipose tissue to clarify the effects of SZ-A on gut microbial communities and BA patterns. Additionally, we investigated the beneficial influence of intestinal barrier proteins and FXR-mediated signaling pathways, focusing on their role in counteracting disruptions in BA and lipid metabolism caused by HFD. These insights were instrumental in identifying the therapeutic potential of SZ-A.

## 2 Materials and methods

### 2.1 Materials

SZ-A powder (J202302021) is characterized by a total polyhydroxy alkaloid content of 54.50%, with specific constituents including 42.66% 1-deoxynojirimycin (DNJ), 7.40% fagomine (FAG), and 4.44% 1,4-dideoxy-1,4-imino-D-arabinitol (DAB). The remaining components are mostly polysaccharides and amino acids (17). This product was graciously provided by Beijing Wuhe Boao Pharmaceutical Co., Ltd. (Beijing, China). Orlistat capsules (Hangzhou Zhongmei East China Pharmaceutical Co., Ltd.) were used. Carboxymethyl cellulose sodium (CMC-Na) (A05925). High-fat feed (MD12032, comprising 45% fat, 20% protein, and 35% carbohydrates) was obtained from Jiangsu Medison Biopharmaceutical Co., Ltd.

The Rat Insulin Competitive ELISA Kit (EK3220-96), Rat Leptin ELISA Kit (EK397-96), Rat IL-1β ELISA Kit (EK301B/3-96), Rat IL-6 ELISA Kit (EK306/3-96), and Rat TNF-A ELISA Kit (EK382/3-96) were obtained from Lianke Biotechnology Co., Ltd. The Rat Glucagon-Like Peptide 1 (GLP-1) ELISA Kit (E-EL-R3007) was obtained from Elabscience Biotechnology Co., Ltd. FXR (25055-1-AP) antibody was purchased from Wuhan Sanying Biotechnology Co., Ltd.; FGF15 (GB114817) antibody; ZO-1 (GB111402) antibody; and occludin (GB111401) antibody. The antibody against claudin-1 (GB112543) was obtained from Wuhan Servicebio Technology Co., Ltd., and the CYP7A1 (A10615) antibody and the TGR5 (A20778) antibody were obtained from ABclonal Biotechnology Co., Ltd.

### 2.2 Animal experiment

Specific pathogen-free (SPF)-grade male Sprague-Dawley (SD) rats, aged 6 weeks and weighing 190–220 g, were used. The research protocol was sanctioned by the Animal Welfare Committee at the Henan Academy of Traditional Chinese Medicine (HNTCMDW-202303011). Housing conditions were maintained at a temperature of 22 ± 2°C, humidity of 45 ± 5%, and light/dark cycle of 12 h/12 h, with unrestricted availability of food and water. Following a 7-d adaptation period, the rats were assigned at random to 2 distinct groups: the normal control group (NCD) and the HFD control group (HFD). Control group rats received a standard diet, whereas HFD control group rats were given HFD for a period of 12 weeks (considered successful modelling if the BW exceeded the normal rat weight by more than 20%). After successful model establishment (rats that did not successfully model were excluded), the successfully modelled rats were divided into 4 groups; HFD control group (Model), SZ-A high-dose group (SZ-A400), SZ-A low-dose group (SZ-A300), and orlistat capsule group (orlistat).

Subsequently, the rats in the control and model groups were gavaged with physiological saline, while those in the SZ-A high-dose group received SZ-A at a dosage of 400 mg/kg/day. Those in the SZ-A low-dose group received SZ-A at a dosage of 300 mg/kg/day. The orlistat group was gavaged with orlistat dissolved in 0.5% CMC-Na at a dosage of 30 mg/kg/day once daily for a continuous period of 6 weeks. During the experimental period, the BWs of the rats were measured weekly. At the conclusion of the study, fecal samples from the rats were collected under aseptic conditions and preserved at a temperature of -80°C.

### 2.3 Serum analysis

After collection, serum was extracted from the blood samples via centrifugation and was then utilized for the quantitative determination of total cholesterol (TC), triglyceride (TG), high-density lipoprotein cholesterol (HDL-C), low-density lipoprotein cholesterol (LDL-C), aspartate aminotransferase (AST), and alanine aminotransferase (ALT) levels using an automated biochemical analyzer.

### 2.4 Histopathological and oil red O analysis

The rat livers were fixed in 10% formalin solution for a period of 48 h, followed by embedding in paraffin. Subsequently, 5-μm-thick sections were prepared. These sections were treated with hematoxylin and eosin (H&E) staining and observed under a light microscope to evaluate the tissue structure. Additionally, rat liver tissue sections were rapidly frozen in liquid nitrogen. Oil Red O staining was conducted on these frozen sections to visualize lipid deposition within hepatocytes, and the stained slides were scrutinized under a microscope to evaluate any pathological alterations indicative of lipid accumulation.

### 2.5 ELISA analysis

The levels of LPS, INS, and GLP-1 in rat serum, as well as the expression levels of the IL-1β, IL-6, and TNF-α in rat small intestine tissue, were determined via ELISA.

### 2.6 Real-time PCR analysis

The tissue was ground thoroughly and centrifuged to obtain the supernatant; 100 μl of alternative chloroform was added; and the mixture was mixed well. After centrifugation, the supernatant was mixed with isopropanol and allowed to stand; the liquid was removed; ethanol was added; and the mixture was inverted for washing. This process was repeated once, and the liquid was completely removed. The mixture was placed on a super clean bench and blown for 3–5 min, and an RNA dissolution solution was added to dissolve the RNA. Complete reverse transcription was performed on a regular PCR machine using the following amplification protocol. The RT-qPCR results were analyzed using a Bio-Rad CFX Connect (Bio-Rad, USA) for fluorescence quantification. The mRNA levels were standardized against GAPDH levels and determined using the ΔΔCT technique (shown in **Table 1**).

**Table 1:** Primer sequences RT-PCR.

Primers	Sequence
mGAPDH	Forward 5′CTGGAGAAACCTGCCAAGTATG 3′ Reverse 5′GGTGGAAGAATGGGAGTTGCT 3′
mZO-1	Forward 5′CGGGCTACCTTATTGAATGTCC3′ Reverse 5′ GAGCGAACTGAATGGTCTGATG3′

### 2.7 Western blot

After the tissue was washed with PBS, it was homogenized and lysed. The supernatant was collected, and electrophoresis and membrane transfer were subsequently performed. The membrane was subjected to overnight incubation with the primary antibody, followed by incubation with the secondary antibody at room temperature. After the unbound antibodies were removed, the PVDF membrane was placed in a chemiluminescence detector. After exposure, the original image was saved in TIFF format.

### 2.8 Immunofluorescence staining

The paraffin sections were deparaffinized and cleaned, immersed in retrieval solution, and heated for antigen retrieval. After blocking with 3% BSA for 30 min, the sections underwent overnight incubation with the primary antibody. After rinsing, the sections were treated with the secondary antibody in darkness for 50 min. Subsequent to washing with PBS buffer, the sections were stained with DAPI, followed by a 10 min incubation period in the dark. API was used to visualize the cell nuclei. Images were obtained using a Zeiss LSM 780 confocal microscope.

### 2.9 Targeted metabolomics study

The BA standard solution was prepared, and the samples were extracted after low-temperature grinding; then the supernatant was collected. The presence and amount of the target compounds in the samples were determined using LC-ESI-MS/MS (UHPLC-Qtrap). The default parameters in the AB SCIEX quantitative software OS were used for automatic recognition and integration of the ion fragments, with manual checks as an auxiliary. Standard curves for linear regression were constructed by plotting the mass spectrometry peak area of the analyte on the y-axis and the analyte concentration on the x-axis. Sample concentration determination: The peak area of the analyte from mass spectrometry was input into the linear regression equation to determine the concentration.

### 2.10 Untargeted metabolomics study

The sample was mixed with 200 μL of precooled 75% methanol, ground for 3 min with a steel ball, vortexed, and extracted on ice via ultrasonication for 3 min. Then, 30 μL of methanol and 600 μL of methyl tert-butyl ether were added, and the mixture was shaken for 30 min. Afterwards, 160 μL of water was added, the mixture was mixed well and allowed to stand for 30 min, and the mixture was centrifuged. After centrifugation, the upper layer was transferred to dryness with nitrogen, after which the sample was reconstituted with 500 μL of a mixture of acetonitrile-isopropanol (CH3CN-IPA, 1:1, V/V) and centrifuged, and the supernatant was collected (the AB SCIEX corporation’s state-of-the-art ultrahigh-performance liquid chromatography hyphenated to a triple quadrupole time-of-flight mass spectrometer, known as the UPLC-TripleTOF system).

### 2.11 Metagenomic sequencing

After DNA extraction from the samples, the genomic DNA extracted was assessed. Fragmentation was performed to an approximate size of 300 bp using a Covaris M220. A paired-end (PE) library was constructed, followed by bridge PCR amplification. Sequencing was then completed on an Illumina HiSeq platform. The data analysis began with the raw sequences obtained from the sequencer. Initially, the raw sequences were subjected to optimization processes, including splitting, quality trimming, and contamination removal. Subsequently, the optimized sequences were used for contig assembly and gene prediction.

### 2.12 Statistical analysis

All the data are presented as the means ± standard deviations (x ± s) and were analyzed using GraphPad Prism 9.5 (San Diego, CA, USA). For multiple group comparisons, one-way analysis of variance and Fisher’s least significant difference *post hoc* test were employed. For comparisons between two groups, an unpaired two-tailed Student’s t-test was used. Statistical significance was determined at p < 0.05 and p < 0.01.

## 3 Results

### 3.1 SZ-A effectively regulates the BW of HFD-induced obese rats

**Figure 1:**
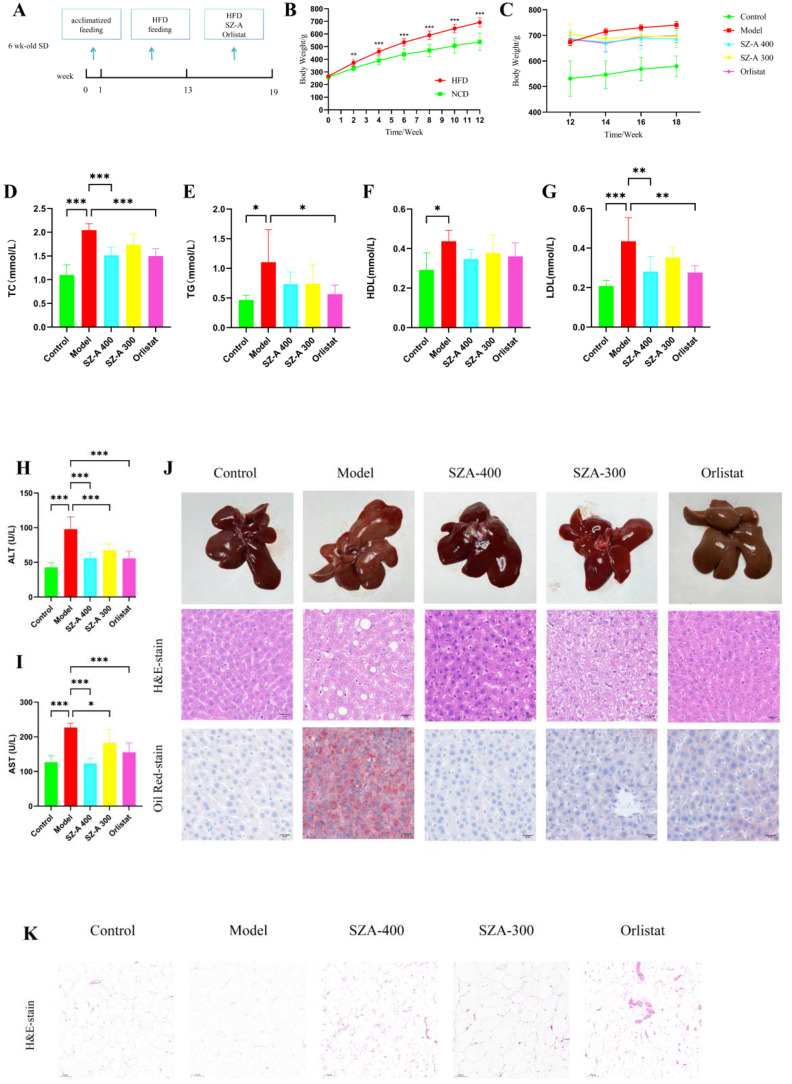
**(A)** Schematic diagram of the experimental procedure (n =10/group). **(B)** Body weight conditions of HFD-induced rats(HFD vs. NCD). **(C)** The effect of SZ-A on the body weight of obese rats (n =7-8/group). **(D-I)** Effect of SZ-A on serum level of TC, TG, HDL, LDL, ALT, AST. (compared to the model group, n = 6). **(J)** The impact of SZ-A on pathological changes in the liver of rats induced by HFD (Scale bar: 20 *µ*m) **(K)** Each group’s epiWAT shows representative H&E staining (Scale bar: 100 *µ*m). (^*^p < 0.05, ^**^p < 0.01, ^***^p < 0.001).

After 1 week of acclimatized feeding, the rats were divided into two groups and fed HFD or a normal diet for 12 weeks, after which SZ-A was administered by gavage (**Figure 1A**). Starting from the second week, there was a notable rise in the BW of the rats on a high-fat diet as opposed to those on a regular diet (P < 0.01). After 12 weeks, the BW of the rats fed by HFD was 692.28 ± 37.5 g, while that of the rats fed a normal diet was 537.38 ± 68.6 g, with the HFD group being more than 20% heavier on average than the normal diet group, indicating a successful modelling of obesity in the rats (**Figure 1B**). Intervention began in the 12th week, and after 6 weeks of intervention, there was a significant reduction in BW among rats treated with either SZ-A or orlistat capsules in the 18th week (P < 0.05). This indicates that SZ-A can effectively suppress weight gain in obese rats (**Figure 1C**).

### 3.2 The impact of SZ-A on blood lipids and liver function in HFD-induced obese rats

Consistent with other studies, we found that SZ-A can effectively ameliorate lipid metabolism disorders and liver function abnormalities. After treatment with SZ-A, the serum levels of TC, LDL, ALT, and AST were notably reduced (P < 0.05) (**Figures 1D–I**). This indicates that SZ-A contributes to a decrease in blood lipid levels and the amelioration of hepatic injury in obese rats.

### 3.3 The impact of SZ-A on pathological changes in the livers and fat accumulation of HFD-fed rats

In the control group, the rat livers exhibited a characteristic reddish-brown hue without surface macules. In contrast, livers from the model group were moderately enlarged, had a soft texture, and presented a yellowish tint with distinct mottling and a greasy texture. The liver appearance of rats treated with SZ-A or orlistat capsules was closer to normal, with minimal yellowing and maculation. H&E staining revealed distinct differences in liver morphology. Livers from the control group displayed well-defined lobular structures with hepatocytes free from fatty degeneration, the absence of discernible fat globules, and an orderly arrangement of cellular nuclei. Conversely, the model group exhibited pronounced hepatocellular ballooning degeneration, intracellular fat vacuoles of varying dimensions, perturbed nuclear organization, and inflammatory necrosis.

Following treatment with SZ-A at both low and high doses or with orlistat capsules, there was a marked reduction in hepatocellular steatosis and ballooning degeneration, as well as a decrease in inflammatory necrotic areas, compared to those in the model group. Staining with Oil Red O exposed a notably increased presence of red-colored lipid droplets in the hepatic tissues of the model group rats, signifying severe fatty liver changes. Treatment with SZ-A or orlistat capsules noticeably ameliorated lipid droplet accumulation. Collectively, these findings suggest that SZ-A effectively mitigates pathological hepatic lipid deposition, inflammation, and fibrotic changes in obese rats (**Figure 1J**).

Subsequently, we evaluated the protective effects of SZ-A on fat accumulation. HFD feeding caused a notable enlargement of adipocytes in the epididymal white adipose tissue (epiWAT) compared to the normal group. Treatment with SZ-A at both low and high doses, as well as orlistat capsules, significantly reduced the adipocyte size in epiWAT compared to the model group. These findings further substantiate that the addition of SZ-A markedly improves fat accumulation in rats subjected to an HFD (**Figure 1K**).

### 3.4 SZ-A modulates the gut microbiota in HFD-fed rats

**Figure 2:**
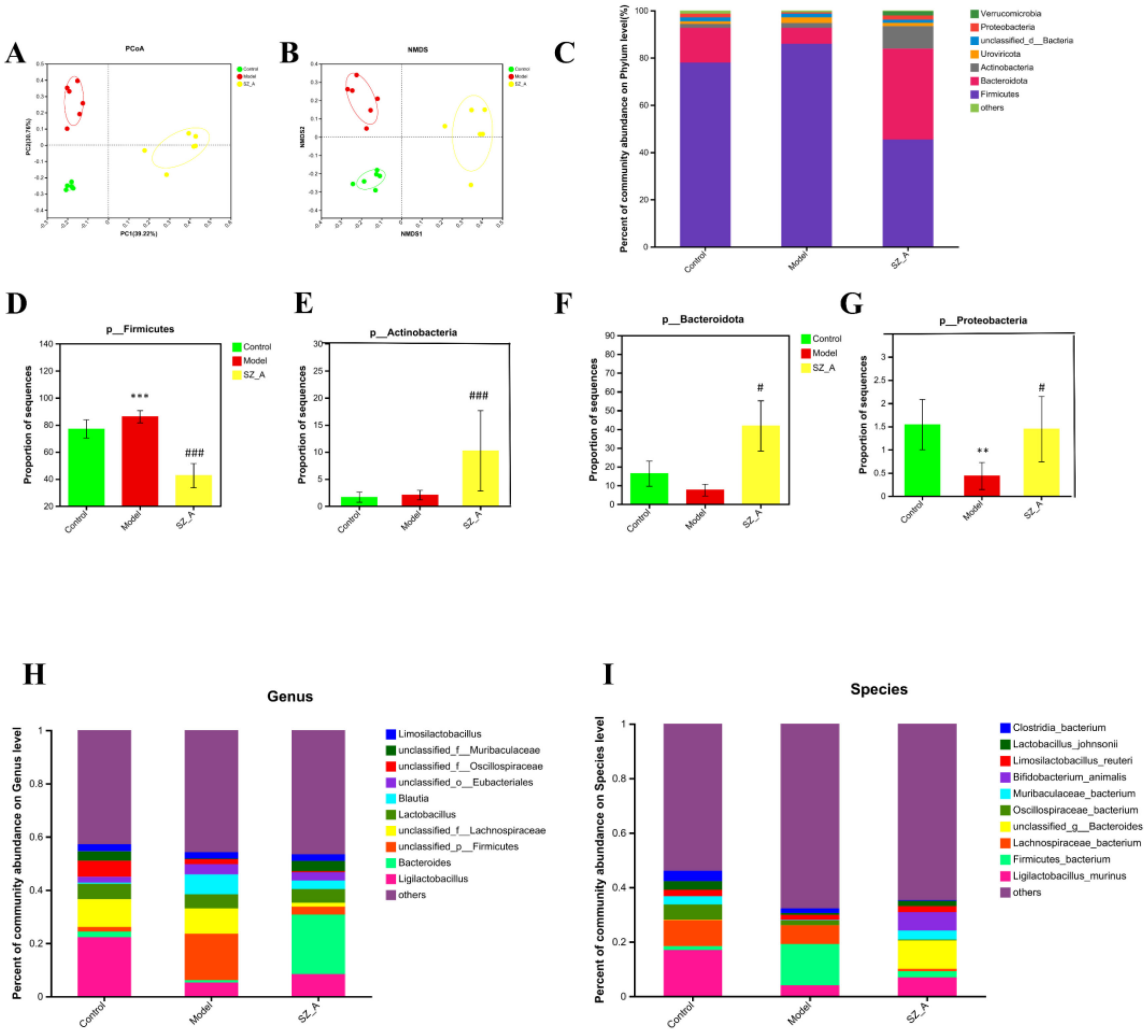
SZ-A modulated the structure and diversity of the gut microbiota (n = 6). **(A)** PCoA score plots at the species level. **(B)** NMDS score plots at the species level. **(C)** Relative abundances of predominant intestinal bacteria at the phylum level. **(D)** The abundance of Firmicutesat the phylum level. **(E)** The abundance of Bacteroidota the phylum level. **(F)** The abundance of Actinobacteriota the phylum level. **(G)** The abundance of Proteobacteria the phylum level (^**^P < 0.01, ^***^P < 0.001 versus control. ^#^P < 0.05, ^###^P < 0.001 versus model). **(H)** taxonomic distribution of bacterial communities at the genus level, **(I)** taxonomic distribution of bacterial communities at the species level.

Intestinal bacteria have a significant association with obesity and imbalances in lipid metabolism; therefore, this study conducted metagenomic sequencing of rat feces to compare the composition of the microbial community in the feces of rats in the control group, model group, and SZ-A. We utilized the abundance of gut bacteria at the operational taxonomic unit level for principal coordinate analysis (PCoA), and the findings highlighted considerable variations in the gut microbial communities across the three rat groups (P = 0.001) (**Figure 2A**). Nonmetric multidimensional scaling analysis indicated that among the three comparisons, the stress value was 0.076 < 0.1, suggesting good ordination, with samples within the same group being closer together, indicating good repeatability within groups and a higher degree of dispersion between different groups. The R value of 0.96, nearing 1, signified greater disparities between groups compared with within-group variations, and P < 0.05 (**Figure 2B**).

### 3.5 SZ-A alleviates HFD-induced gut microbiota dysbiosis

Based on the NR species annotation results, the dominant species in the rat communities at the phylum level were analyzed for different groups. The community bar chart shows the changes in the composition of the dominant species (**Figure 2C**), with the dominant bacterial groups in rat feces being Firmicutes, Bacteroidota, and Actinobacteriota. In contrast to the control group, the model group exhibited a markedly reduced presence of Proteobacteria in their fecal bacteria (P < 0.01), whereas the SZ-A group showed a significant increase in both Actinobacteria and Bacteroidota (P < 0.01, P < 0.001), and the relative abundance of Firmicutes was obviously lower (P < 0.001). As opposed to the model group, the SZ-A group’s fecal microbiota showed a notably higher relative abundance of Actinobacteria and Proteobacteria (P < 0.05). Additionally, there was a notable rise in the relative abundance of Bacteroidota (P < 0.001) and a corresponding significant decrease in Firmicutes (P < 0.001). At the phylum level, compared with those in the control group, the ratio of Firmicutes to Bacteroidota in the model group was severely disrupted, with a notable rise in the abundance of Firmicutes and a corresponding decline in the abundance of Bacteroidota. SZ-A is capable of modulating the proportion of these bacterial groups to resemble the control group’s community composition. The Firmicutes to Bacteroidota ratio serves as an indicator of the gut microbiota’s equilibrium, with a reduced Firmicutes/Bacteroidota ratio being directly associated with weight reduction (**Figures 2D–G**). Our subsequent analyses at the genus and species levels revealed significant changes in the gut microbiota among the groups. At the genus level, in contrast to the control group, the relative abundance of *Blautia* increased, while that of *Ligilactobacillus* significantly decreased in the model group. The SZ-A group exhibited a notably higher relative abundance of *Bacteroides* and a considerably lower relative abundance of *Blautia*, in contrast to the model group. At the genus level, the abundances of *Bifidobacterium animalis*, *Ligilactobacillus murinus*, and *Muribaculaceae* bacterium increased in the SZ-A group, while the abundances of Firmicutes bacterium and Lachnospiraceae bacterium decreased. These results indicate that SZ-A directly influences the modulation of gut microbiota composition in rats with HFD (**Figures 2H–I**).

**Figure 3:**
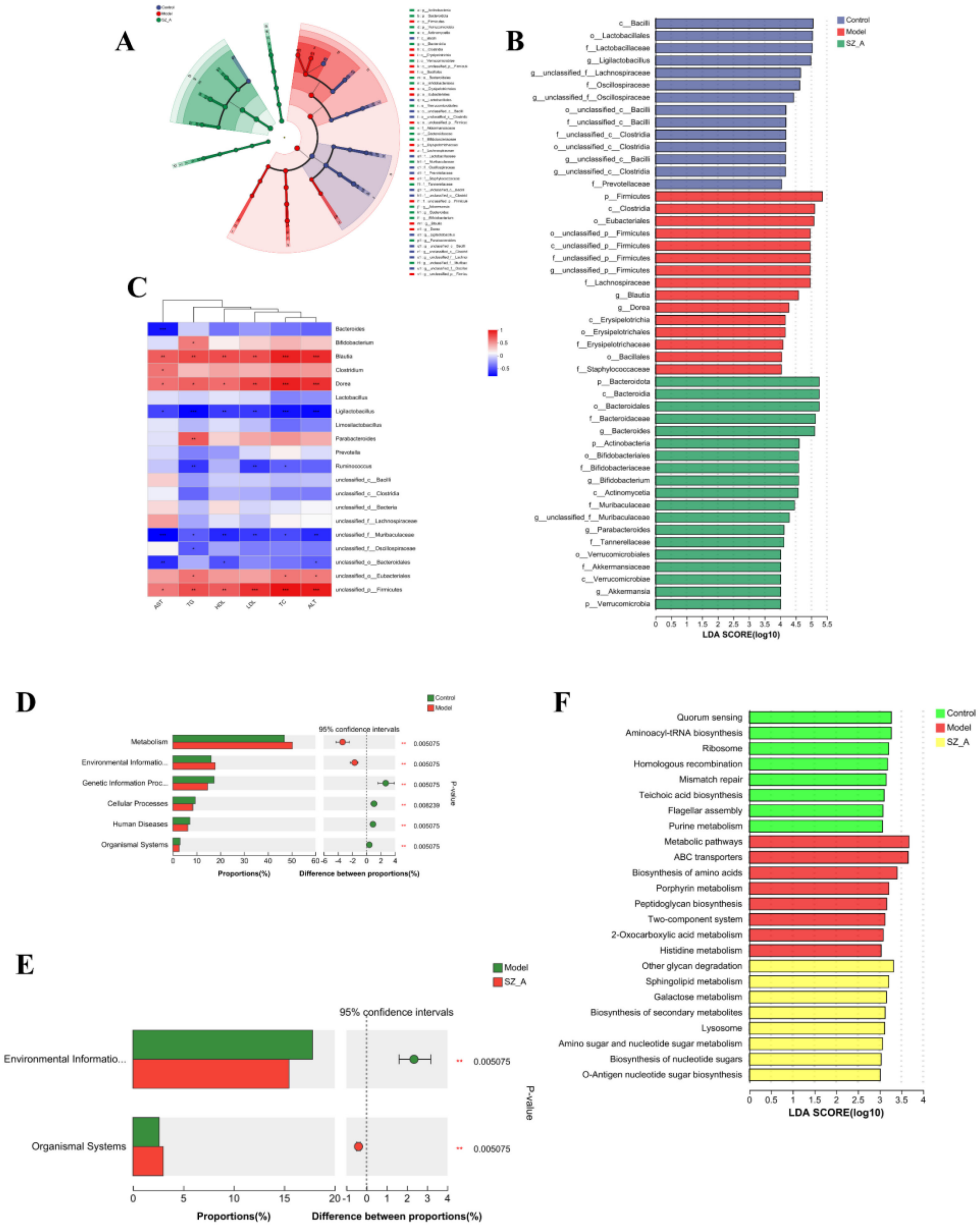
**(A)** LefSe analysis of the rat gut microbiota. **(B)** LDA distribution. **(C)** Heatmap of Spearman correlation analysis between the characteristic gut microbiota (genus) and blood lipids, liver function (n = 6). **(D–E)** KEGG analysis of microbial community functions in the feces. **(F)** LefSe Analysis of KEGG. ^*^P ≤ 0.05, ^**^p ≤0.01, ^***^p ≤ 0.001 .

Using the LEfSe (LDA>4.0) method, statistical analysis of the relative abundance of gut microbiota of rats in each group was performed to identify group-specific and representative differential species, resulting in an evolutionary branch diagram (**Figure 3A**) and a statistically significant biomarker abundance diagram (**Figure 3B**). There were 14 trees in the control group, 15 in the model group, and 19 in the SZ-A group, and these branches had different abundances and were considered characteristic bacterial phenotypes. The result indicated a notable change in microbial abundance under the intervention of HFD and SZ-A. Among them, Oscillospiraceae (Firmicutes) was a characteristic genus in the control group. In the model group, *Blautia* (genus) and Firmicutes (phylum) were characteristic genera. In the SZ-A group, *Bacteroides* (genus), Bacteroidaceae (Firmicutes), Bifidobacteriaceae (Firmicutes), *Bifidobacterium* (genus), and *Bifidobacteriales* (order) were characteristic genera.

Spearman correlation analysis assessed the relationship among the predominant 20 bacterial genera and the rats’ blood lipid levels and hepatic function indices. *Blautia* and *Dorea* showed positive associations with blood lipids and liver health biomarkers, whereas *Ligilactobacillus* exhibited an inverse relationship with these parameters. The abundance of *Bifidobacterium* and *Parabacteroides* was positively associated with TG levels. The abundance of *Bacteroides* was negatively associated with AST levels, and that of *Ruminococcus* had negative correlations with TG levels, TC levels, and LDL levels. Spearman correlation analysis indicated that these bacteria were closely related to parameters associated with hyperlipidemia (**Figure 3C**).

We also conducted functional analysis using the KEGG database to predict the functional pathways associated with gut microbiota. The control group’s gut microbiota displayed enhanced capabilities in genetic information processing, cellular activities, human diseases, and organismal systems, while that of the model group showed an increase in metabolism and environmental information processing. The SZ-A group experienced a notably reduced capacity for environmental information processing relative to the model group (**Figures 3D, E**). To screen for differential metabolic functional pathways, we performed LEfSe analysis of the KEGG metabolic pathways. The results indicated that functional name, mismatch repair, ribosome, flagellar assembly, purine metabolism, aminoacyl-tRNA biosynthesis, homologous recombination, teichoic acid biosynthesis (phospholipid biosynthesis), and quorum sensing were significantly enriched in the control group. Peptidoglycan biosynthesis, porphyrin metabolism, two-component system, metabolic pathways, ABC transporters, biosynthesis of amino acids, 2-oxocarboxylic acid metabolism, and histidine metabolism were notably enriched in the model group. Sphingolipid metabolism, lysosome, amino sugar and nucleotide sugar metabolism, biosynthesis of nucleotide sugars, galactose metabolism, other glycan degradation, O-antigen nucleotide sugar biosynthesis, and biosynthesis of secondary metabolites were significantly enriched in the SZ-A group (**Figure 3F**).

**Figure 4:**
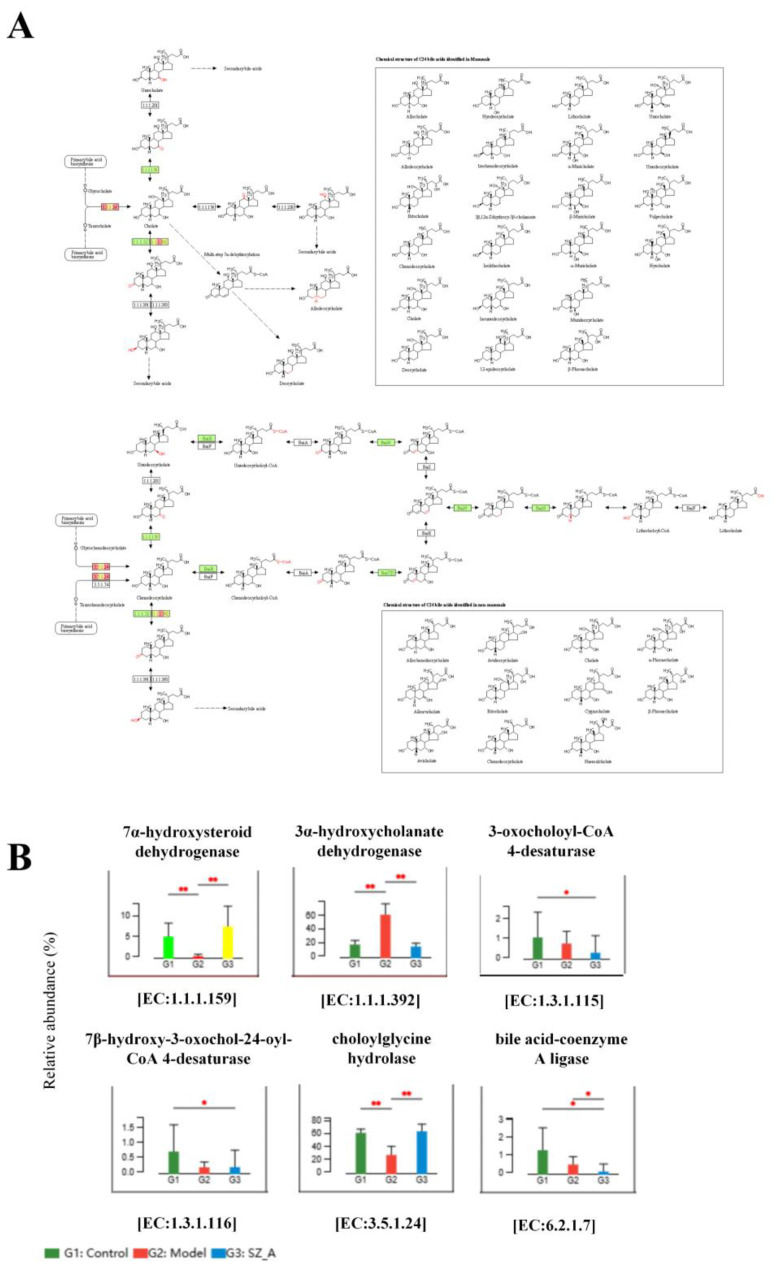
KEGG pathway enrichment analysis of secondary BA synthesis pathway. **(A)** Metagenomic analysis of inter-population differences in secondary bile acid metabolism pathways (red represents enriched genes, green represents no significant difference between the two groups) **(B)** Metabolic pathway inter-group difference test. ^*^P ≤ 0.05, ^**^p ≤0.01.

Based on the KEGG annotation results, we conducted a differential significance analysis of enzymes involved in the secondary BA pathway. The KEGG gene enrichment analysis indicated that, compared with those in the model group, 7α-hydroxysteroid dehydrogenase (7α-HSDH) and choloylglycine hydrolase were significantly enriched in both the control group and the SZ-A group. 7α-HSDH plays an important role in bile acid (BA) metabolism, efficiently converting taurochenodeoxycholic acid (TCDCA) into tauroursodeoxycholic acid (TUDCA). Choloylglycine hydrolase, also known as bile salt hydrolase or conjugated BA hydrolase, is an enzyme that plays an important role in BA metabolism. Conversely, the concentration of 3α-hydroxycholanate dehydrogenase(3α-HSDH) was markedly reduced in both the control and SZ-A groups as compared with the model group. This finding suggested that HFD-induced obesity can affect the composition of gut microbiota through the regulation of BAs (**Figures 4A, B**).

### 3.6 Lipidomic analysis of HFD-fed rats

**Figure 5:**
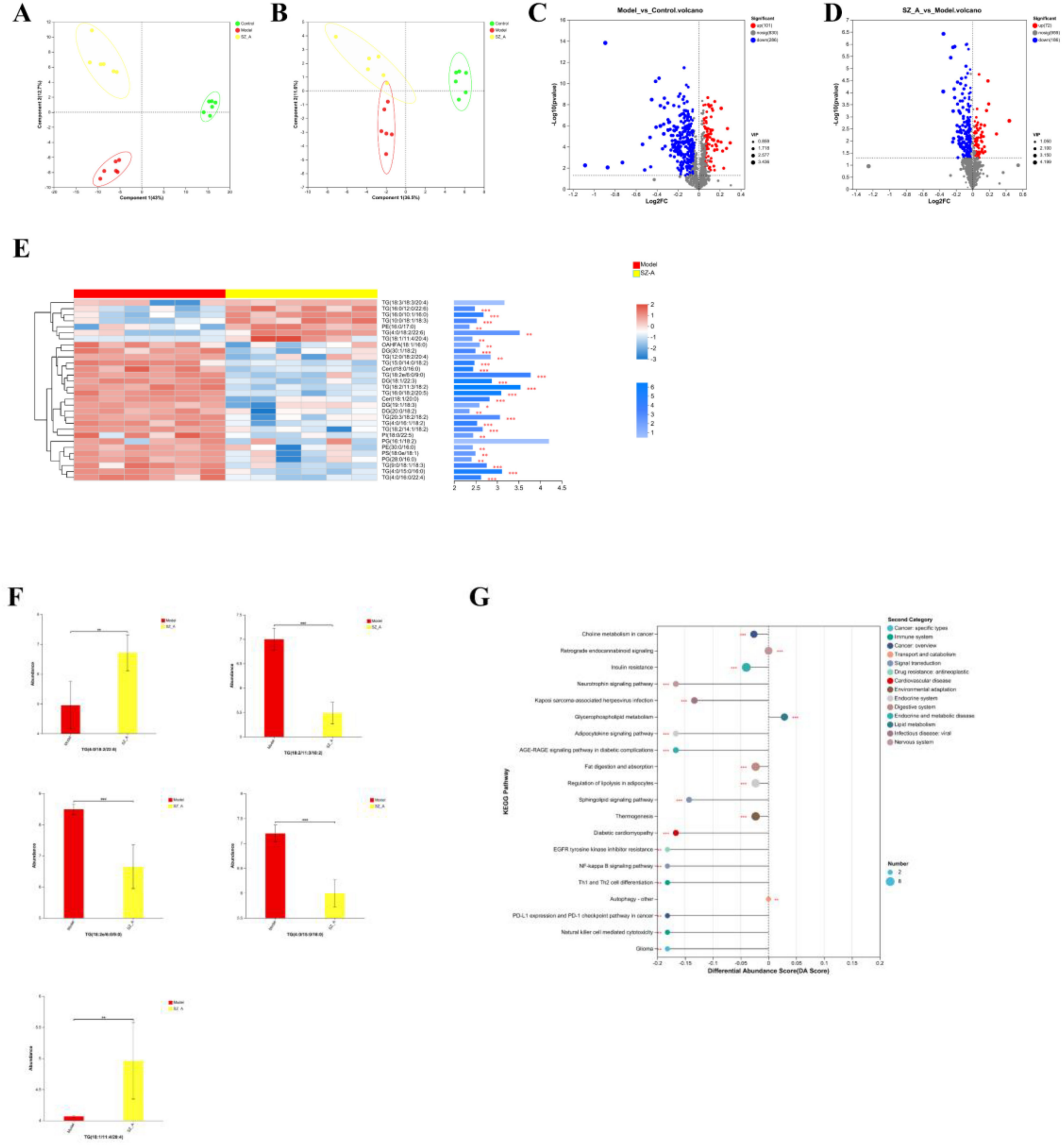
**(A, B)** PLS-DA analysis of rat adipose tissue, **(C, D)** Volcano plot of adipose tissue. **(E)** Top 30 VIP values of the model group and the SZ-A group. **(F)** differential lipidomics analysis between the model group and the SZ-A group. **(G)** KEGG pathway differential abundance score plot.*P ≤ 0.05, **p ≤0.01, ***p ≤ 0.001.

For a comprehensive grasp of SZ-A’s impact on lipid and metabolic processes, we used lipidomics methods to detect differences and changes in lipids in rat adipose tissue. We identified 607 lipid metabolic products from rat adipose tissue and performed PLS-DA on the samples. Under the positive and negative modes, there was a clear distinction in lipid grouping among the control group, model group, and SZ-A, indicating notable differences in fat lipid metabolism among the groups (**Figures 5A, B**).

Student’s t-test was applied to analyze lipid metabolites, and differentially abundant metabolites were screened with the criteria of a fold change (FC) ≥1 and P < 0.05. Overall, the comparison between the control group and the model group revealed a total of 387 distinct metabolic compounds, with 286 experiencing an increase in abundance and 101 showing a decrease. The main changes were the upregulation of triglycerides (TGs) and diglycerides (DGs), as well as the downregulation of phosphatidylcholine (PC) and phosphatidylethanolamine (PE). Between the model group and SZ-A, 258 differentially abundant metabolites were detected, with 72 upregulated and 186 downregulated. This indicates that SZ-A is involved in modulating lipid metabolism in rats with obesity (**Figures 5C, D**).

Next, we utilized OPLS-DA model analysis to obtain the top 30 VIP scores, among which TG (18:2e/6:0/9:0), TG (18:2/11:3/18:2), TG (4:0/15:0/16:0), TG (4:0/18:2/22:6), and TG (18:1/11:4/20:4) were the top 5 lipid components contributing to the separation between the model group and SZ-A. These are lipid subclasses within the glycerolipid (GL) category and play a key role in improving lipid metabolism disorders in obese rats (**Figures 5E, F**).

For KEGG enrichment analysis of differentially enriched adipose tissue between the model group and the SZ-A group, we selected the top 13 metabolic function categories with significant relevance, which were mainly enriched in the following 13 pathways: choline metabolism in cancer, insulin resistance, neurotrophin signaling mechanism, Kaposi’s sarcoma-associated herpesvirus infection, glycerophospholipid metabolism, retrograde endocannabinoid signaling, adipocytokine signaling pathway, AGE-RAGE signaling mechanism in diabetic complications, fat digestion and absorption, regulation of lipolysis in adipocytes, sphingolipid signaling mechanism, thermogenesis, and diabetic cardiomyopathy (**Figure 5G**).

### 3.7 SZ-A regulates the profiles of BA metabolites

**Figure 6:**
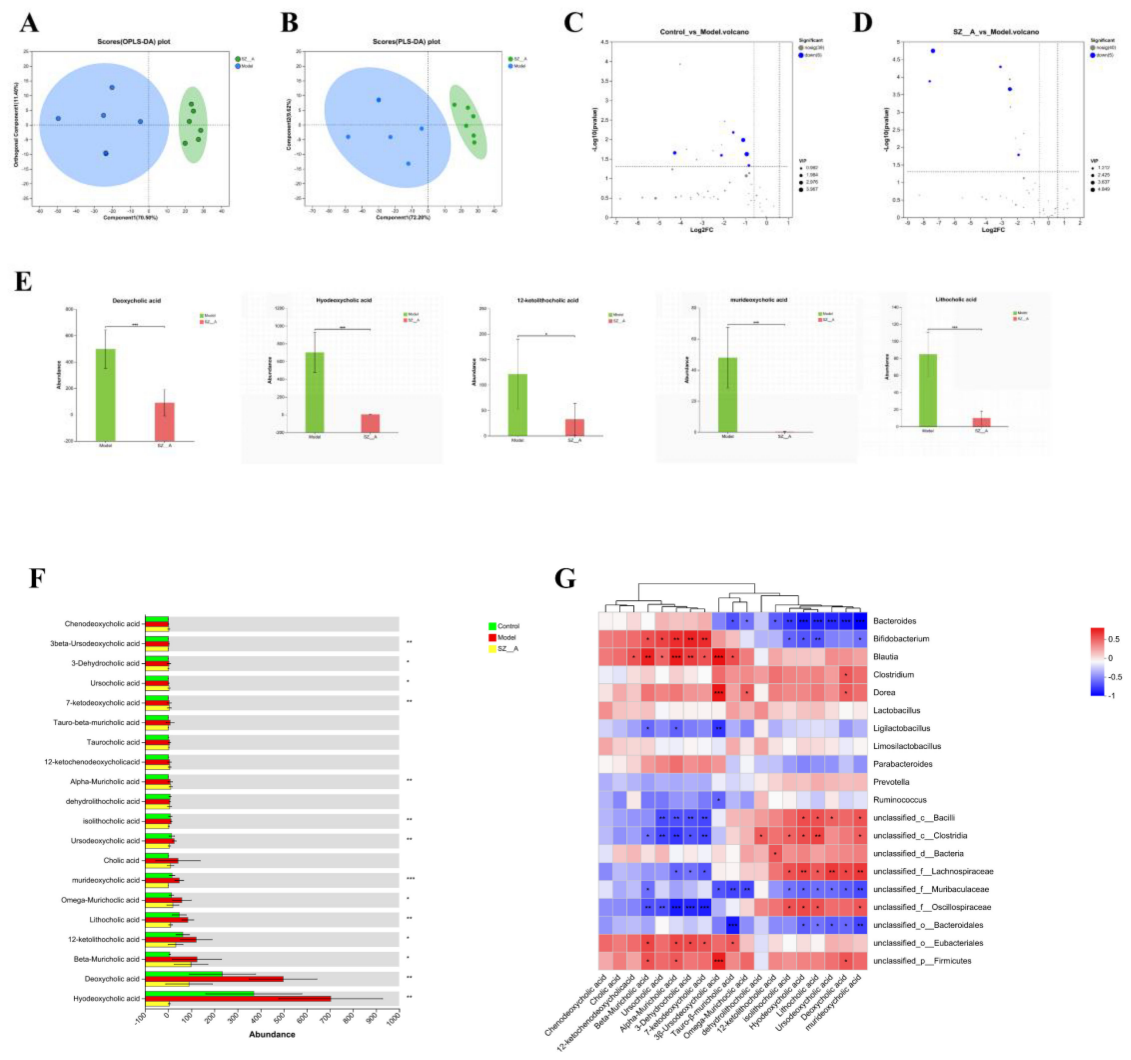
Effect of SZ-A treatment on fecal BA metabolites in HFD-fed rats. **(A)** PLS-DA score plot in the positive mode. **(B)** PLS-DA score plot in the negative mode. **(C, D)** Volcano plot of BA metabolites. **(E)** differential BA metabolites analysis between the model group and the SZ-A group. **(F)** The quantitative abundance of BAs was observed significantly altered in different groups. **(G)** Correlation heatmap between rat fecal bile acids and the relative abundance of characteristic gut microbiota (genus). The color gradient represents the correlation coefficient, with red indicating more positive and bluer indicating more negative. ^*^p < 0.05, ^**^p < 0.01, ^***^p < 0.001.

We utilized BA-targeted metabolomics to measure the BA content in rat feces. The results showed that the BA metabolic profiles of the model group and the SZ-A group were significantly different, with a greater degree of separation between the two groups, indicating a notable classification effect and significant differences (**Figures 6A, B**). Student’s t-test was applied to analyze BAs in rat feces, and differentially abundant metabolites were screened using the criteria of FC ≥ 1.5, P < 0.05, and a variable importance (VIP) > 1.0 in the OPLS-DA model between the two groups (**Figures 6C, D**). The levels of hyodeoxycholic acid (HDCA), deoxycholic acid (DCA), 12-ketolithocholic acid (12-KLCA), lithocholic acid (LCA), and murideoxycholic acid (MDCA) significantly decreased in the SZ-A group (**Figures 6E, F**).

Spearman’s correlation analysis was employed to assess the relationship between the 20 predominant bacterial genera and the 20 key BA metabolite genera identified in rat fecal samples. The study revealed that murideoxycholic acid, deoxycholic acid, ursodeoxycholic acid, lithocholic acid, hyodeoxycholic acid, and isolithocholic acid were negatively correlated with BAs, such as *Bacteroides*. Ursodeoxycholic acid (3β-ursodeoxycholic acid), 7-ketodeoxycholic acid, 3-dehydrocholic acid, beta-muricholic acid, alpha-muricholic acid, and ursocholic acid were positively correlated with *Bifidobacterium* and *Blautia* (**Figure 6G**).

### 3.8 SZ-A mitigates obesity and lipid metabolism disruptions by modulating the BA signaling pathway

In contrast to the control group, the model group exhibited a marked increase in the levels of FXR and FGF15 proteins within the rat small intestine (P < 0.01), while the CYP7A1 content was notably decreased (P < 0.01). This indicates that obesity triggers the activation of the FXR/FGF15 signaling pathway in the rat small intestine, which coincides with a pronounced decrease in hepatic CYP7A1 levels (P < 0.01). Relative to the model group, the small intestinal FXR and FGF15 levels in the rats in both the low- and high-dose SZ-A groups, as well as those in the orlistat group, were notably lower (P < 0.05, P < 0.01). Additionally, the protein expression of CYP7A1 was substantially upregulated (P < 0.01). These results indicate that SZ-A can effectively counteract obesity-induced activation of the FXR/FGF15 signaling pathway in rats and suppress CYP7A1 expression.

**Figure 7:**
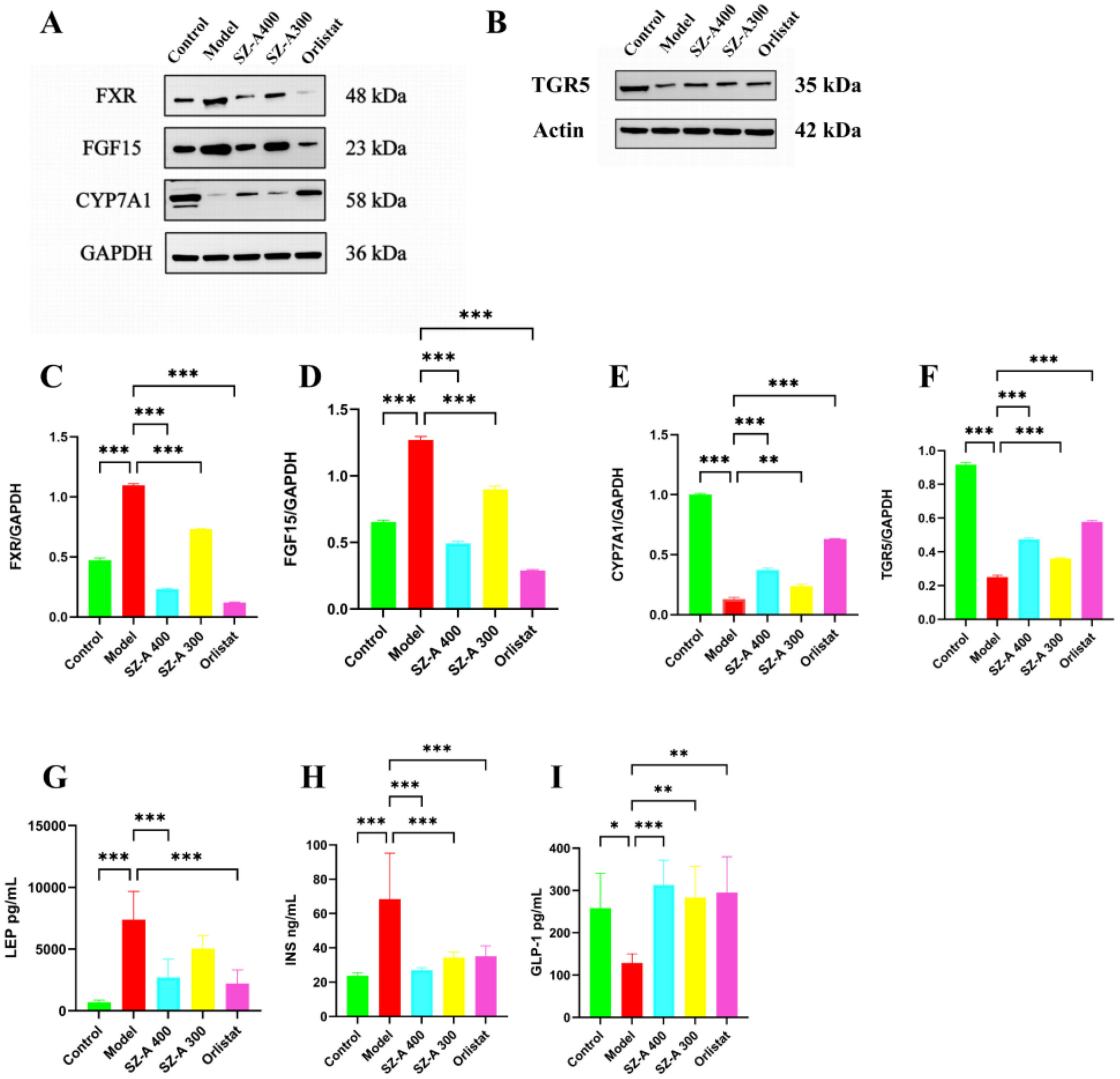
SZ-A could regulate the expression of lipid-related proteins **(A, B)** The expression of lipid-related proteins of FXR, FGF15, CYP7A1, TGR5, by Western blot. **(C–F)** The protein level of FXR, FGF15, CYP7A1, TGR5 were examined by Western blots. **(G–I)** Determine the levels of LEP, INS, and GLP-1 in rat serum using an ELISA kit.*P ≤ 0.05, **p ≤0.01, ***p ≤ 0.001.

Relative to the control group, the TGR5 protein content in the small intestine of rats in the model group was notably reduced (P < 0.01), suggesting that SZ-A can notably suppress the activation of TGR5 in the rat small intestine caused by obesity and inhibit the expression of TGR5 (**Figures 7A–F**).

### 3.9 The impact of SZ-A on LEP, INS, and GLP-1 levels in the serum of HFD-induced obese rats

Relative to the control group, the LEP levels in the model group were greater (P < 0.001). Relative to the model group, both the high-dose SZ-A group and the orlistat group showed a reduction in serum LEP levels, reflecting an increase in leptin sensitivity (P < 0.001). We further measured serum insulin levels and GLP-1 activity levels as indicators of insulin and GLP-1 secretion, respectively. Compared to those in the control group, the model group rats had increased insulin levels (P < 0.001). Relative to the model group, both the high- and low-dose SZ-A groups and the orlistat group showed a decrease in serum insulin levels, reflecting an increase in insulin sensitivity (P < 0.001). Relative to the control group, GLP-1 levels were lower in the model group (P < 0.05). Relative to the model group, both the high- and low-dose SZ-A groups and the orlistat group had increased serum GLP-1 levels (P < 0.01) (**Figures 7G–I**).

### 3.10 SZ-A alleviated mild, ongoing inflammation within the intestines and bolstered the completeness of the intestinal barrier in rats that were given HFD

**Figure 8:**
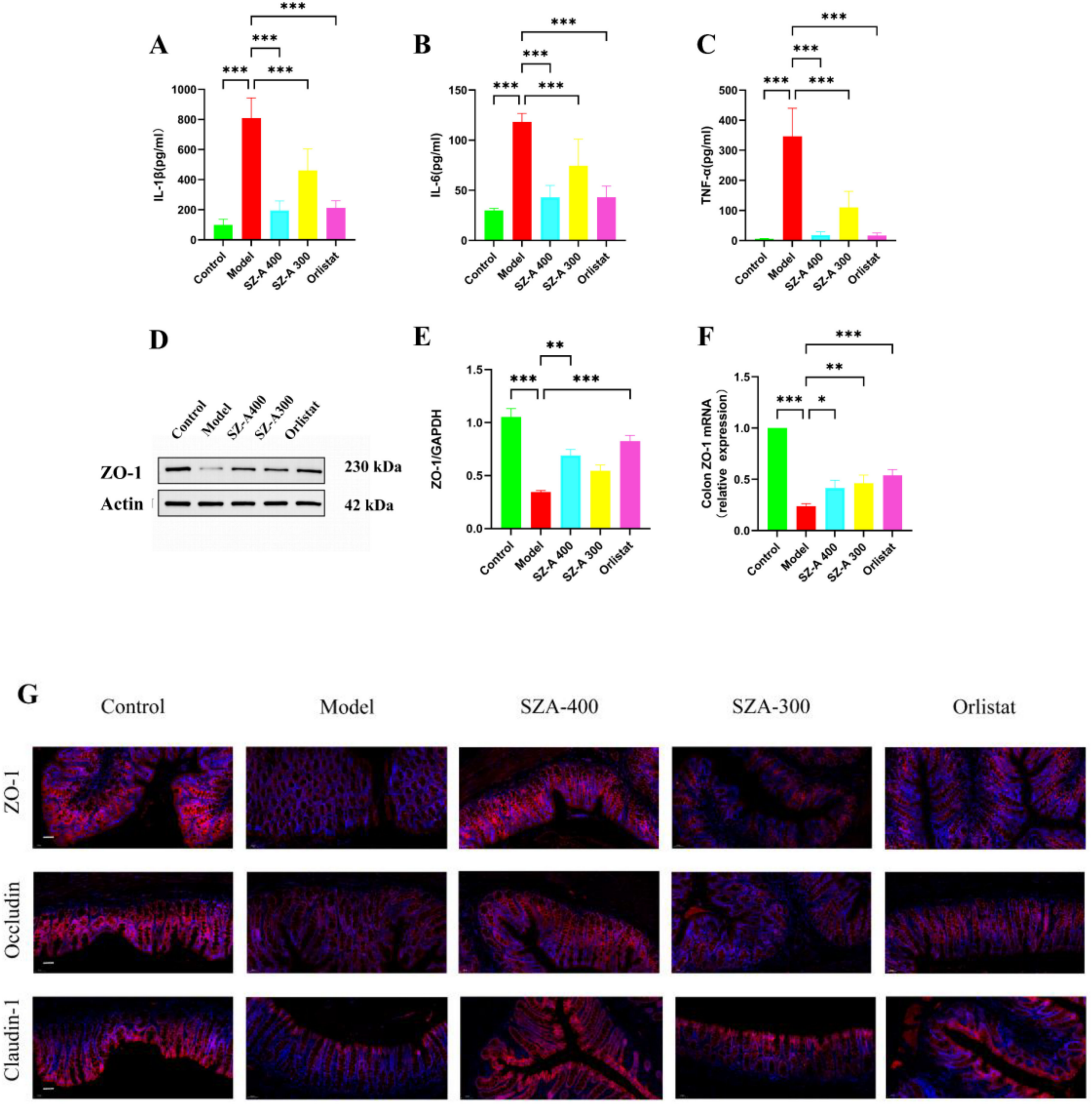
SZ-A can restore the impaired intestinal barrier and improve low-grade intestinal inflammation in rats fed a high-fat diet. **(A-C)** The ELISA kits were used to measure the levels of IL-1β **(A)**, IL-6 **(B)** and TNF-α **(C)** in the ileum. **(D)** The expression of lipid-related protein of ZO-1 by Western blot. **(E)** The protein level of ZO-1 was examined by Western blots. **(F)** Relative mRNA levels of ZO-1 in the colon were detected by Real time-PCR analysis. **(G)** Representative picture of immunofluorescence of ZO-1, claudin-1 and occludin (Scale bar: 50 *µ*m) (^*^P < 0.05, ^**^P < 0.01, and ^***^P < 0.001, compared to the model group).

To further investigate the impact of SZ-A on intestinal inflammation in obese rats, we measured the levels of inflammatory factors in the colonic tissues. Relative to the model group, the levels of IL-1β, IL-6, and TNF-α in the colonic tissues of rats in the low- and high-dose SZ-A groups and the orlistat group were notably reduced (P < 0.01); therefore, SZ-A can alleviate intestinal inflammation in obese rats (**Figures 8A–C**).

Under normal physiological conditions, an intact intestinal mucosal barrier can prevent the permeation of bacteria and their mediators into the systemic circulation. Epithelial tight junctions (TJs) are composed of ZO-1, occludin and claudin-1 among others. These tight junction complexes effectively seal intercellular spaces, preventing the invasion of pathogens, endotoxins, and bacteria. Relative to the model group, the protein and mRNA expression of ZO-1 in the SZ-A group increased (**Figures 8D–F**). Our research results indicate that compared to the model group, SZ-A can regulate the expression of ZO-1, occludin, and claudin-1 staining in TJs, thereby maintaining the integrity of the intestinal barrier (**Figure 8G**).

## 4 Discussion

Obesity, a chronic metabolic disorder, arises from an overabundance of energy intake causing a disruption in energy balance and excessive fat accumulation. Recent research has delved deeper into the intricate relationships among the gut microbiota, BAs, and their connection to obesity and lipid metabolic disorders, with an increasing body of evidence underscoring the pivotal role of gut microbiota imbalance and BA metabolic dysregulation in the onset and progression of obesity. Previous research has indicated that SZ-A can promote weight reduction in HFD-fed rats by enhancing the abundance of gut microbiota and modulating metabolic pathways involving amino acids, unsaturated fatty acids, and BAs (18). Our study used metagenomic sequencing, lipidomics, and targeted BA metabolomics to further elucidate the impact of SZ-A on BA metabolism and the gut microbiota. This study revealed the antiobesity effects of this compound through the activation of the TGR5 and FXR signaling pathways and its capacity to mitigate obesity and lipid metabolism issues by enhancing intestinal barrier dysfunction and reducing intestinal inflammation.

A burgeoning array of studies underscores the latent influence of gut microbiota shifts on obesity (19, 20). The consumption of high-calorie diets diminishes the population of beneficial intestinal bacteria, such as lactobacilli, thereby undermining the inherent defense mechanisms of the gut (21). A pivotal difference in the gut microbiome between obese and healthy individuals lies in the altered proportions of the phyla Firmicutes and Bacteroidetes, which collectively constitute around 90% of the adult gut microbiota (22). The interplay between Bacteroidetes and Firmicutes, the two predominant phyla in fecal microbiomes, has greater implications for obesity and associated conditions. For example, an elevated Firmicutes/Bacteroidetes ratio is observed in obese individuals, with an inverse trend observed as caloric intake decreases or weight loss occurs (23). Metagenomic and biochemical assessments of the distal gut microbiota in genetically obese mice have corroborated a marked reduction in Bacteroidetes and an increase in Firmicutes (24). The modulation of specific bacterial populations, characterized by an increase in Bacteroidetes and a decrease in Firmicutes, may offer therapeutic benefits for obesity management (25). The genus *Bacteroides* is essential for maintaining probiotic balance in the human GI tract (26). As gram-negative, non-spore-forming, obligate anaerobic rods, *Bacteroides* exhibit resistance to BAs and proliferate within the gut, conferring benefits to the host (27). Evidence suggests that *Bacteroides* is a probiotic inversely related to obesity, with increased abundance posttreatment potentially halting the progression of obesity (28). A significant reduction in *Bacteroides* abundance is noted among obese individuals (29), a finding that resonates with our results in which a notable decrease in *Bacteroides* was observed in SZ-A relative to obese rats. Additionally, the outer membrane vesicles (OMVs) of *Bacteroides* may contribute to both human health and disease. Hydrolytic enzymes within OMVs play a crucial role in the gut microbial ecosystem. The constituents of OMVs aid in the breakdown of complex polysaccharides, proteins, and lipids, thereby supporting the growth of other bacteria and maintaining intestinal homeostasis (30). Our experimental findings align with these insights, revealing a significant increase in the relative abundance of Bacteroidetes and a pronounced reduction in Firmicutes in response to treatment with SZ-A, suggesting that weight reduction can be achieved through the modulation of these bacterial ratios. The increase in *Bacteroides* can enhance the intestinal barrier, thereby maintaining gut homeostasis, reducing inflammation, and contributing to the stability of the immune system. In summary, the relationship between *Bacteroides* and human health is multifaceted, encompassing nutritional metabolism, immune regulation, maintenance of the intestinal barrier, and associations with obesity and metabolic diseases. Further exploration of *Bacteroides* may yield therapeutic benefits for the management of obesity.

*Blautia* is ubiquitous in the feces and intestines of mammals, contributing to the amelioration of inflammatory and metabolic diseases (31, 32). Studies have indicated that *Blautia* is negatively correlated with visceral fat area, which is considered a biomarker of obesity associated with cardiovascular and metabolic disease risks. In our research, *Blautia* was significantly reduced in obese rats, but treatment with SZ-A increased its population. *Blautia* has great potential for regulating host health and alleviating metabolic syndrome, and further investigation into *Blautia* could hold promising prospects for the treatment of obesity. The majority of studies have established a positive association between *Dorea* and BMI as well as waist circumference (33, 34). Dietary adjustments have been shown to mitigate the impact of detrimental bacteria, such as *Dorea*, thereby curbing obesity induced by HFD in rats (35). Moreover, the enrichment with *Dorea* bacteria is considered to have pro-inflammatory effects (36). Our study corroborates these findings, indicating a notably elevated presence of *Dorea* in HFD-fed rats, with a marked decrease following treatment with SZ-A. Therefore, reducing the proliferation of harmful bacteria and maintaining the stability of the gut microbiota are even more crucial for health.

The development of obesity is closely related to lipid metabolism and inflammatory responses. To gain a deeper understanding of lipid changes in an obese state, we conducted an untargeted lipidomics study. At the metabolomic level, the primary metabolic pathways for SZ-A and the model group encompass a variety of lipid metabolisms, including fatty acyls (FA), glycerolipids (GL), and glycerophospholipids (GP). Dysregulation of lipid metabolism is a major cause of obesity. SZ-A was able to upregulate the levels of TG (4:0/18:2/22:6) and TG (18:1/11:4/20:4), while downregulating the levels of TG (18:2e/6:0/9:0), TG (18:2/11:3/18:2), and TG (4:0/15:0/16:0), thereby improving the metabolism of GL. GL play a significant role in the onset and progression of obesity, not only because it affects the accumulation of triglycerides in adipose tissue but also because it involves disruptions in lipid metabolism and the regulation of energy balance (37). Studies have shown that there is a correlation between glycerolipid metabolism and the gut microbiota regulated by HFD, which is involved in the development of hyperlipidemia (38). Based on lipidomics analysis, further studies are needed to verify the effects of SZ-A on gut microbiota and lipid metabolism. Exploring the impact of these key metabolites on obesity should be a priority in future work.

BAs, pivotal metabolites of cholesterol, are secreted from the liver into the small intestine as bile, where they are metabolized by gut bacteria, reabsorbed by the liver, and enter a cycle of reuse, which aids in the absorption of fats and fat-soluble vitamins (39). Additionally, BAs have the capacity to modulate BW by influencing metabolic and energy balance mechanisms. Research has revealed disparities in the gut microbiota between obese and nonobese individuals, with these differences being linked to BA metabolism. Notably, the gut microbiota in obese individuals is less diverse, and certain strains can suppress the synthesis and absorption of BAs, leading to diminished BA levels that impact fat metabolism and energy balance, thus culminating in weight gain. An imbalance in the gut microbiota can disrupt the metabolism and conversion of BAs, thereby altering their concentration and composition and precipitating metabolic disarray (40). HDCA, a type of secondary BA generated by the gut microbiota in the small intestine, has shown an inverse relationship with the occurrence and severity of non-alcoholic fatty liver disease (NAFLD) across various mouse models, and its alleviating effect on NAFLD has been attributed to the inhibition of intestinal FXR (41, 42). HDCA influences cholesterol metabolism through a variety of mechanisms, including regulating intestinal absorption, affecting BA metabolism, modulating through the gut–liver axis, and promoting cholesterol efflux. These actions play a significant role in maintaining cholesterol homeostasis and in preventing and treating related metabolic diseases (43). Our results highlight a marked increase in HDCA levels in the fecal BAs of obese rats, which were significantly reduced in rats treated with SZ-A. Obesity induced by diet or genetics can trigger alterations in the gut microbiota, leading to elevated DCA levels (44). Our study identified a notably elevated presence in deoxycholic acid in the fecal BA of obese rats, which was notably diminished in the group administered SZ-A. HFD is known to elevate the levels of secondary BA LCA (45). Correspondingly, our study revealed a pronounced increase in LCA in the fecal BAs of obese rats, with a significant decrease observed following treatment with SZ-A. 12-KLCA, through its role in BA metabolism, as a biomarker of kidney injury, and its impact on the gut microbiota and immune responses, may be associated with the development of fat metabolism and obesity. However, further research is needed to clarify the specific mechanisms and causal relationships between 12-KLCA and obesity (46). BAs rely on the metabolic capabilities of the gut microbiota while also influencing the structure and function of microbial communities. BAs and gut microbes interact and affect each other in metabolic diseases. Our experiments also revealed that SZ-A regulates metabolic disorders in obese rats by modulating the BA metabolic pathway. BAs play a significant role in the onset and progression of obesity, but the specific molecular mechanisms and signaling pathways require further research for elucidation.

Imbalances in the gut microbiota that alter BA metabolism and modulate the FXR/TGR5 signaling pathways may contribute to the progression of obesity. In recent years, the gut microbiota and BA signaling pathways have emerged as potential therapeutic targets for combating obesity-related conditions. Changes in the composition or activity of the gut microbiota induced by factors such as antibiotics, alterations in physical activity, diet, or other factors can disrupt BA metabolism (34). Gut bacteria influence obesity by mediating shifts in the BA spectrum. Metabolites produced by these bacteria can alter the body’s metabolism through their respective receptors, promoting processes such as deconjugation, dehydrogenation, and dehydroxylation of primary BAs in the distal small intestine and colon. This increases the chemical diversity of BAs and significantly impacts BA metabolism. BAs produced in the liver undergo secondary metabolism influenced by gut bacteria, which can change the composition of the BA pool. In turn, BAs can affect the gut microbiota through antimicrobial activity and indirect signaling pathways (47). It has been reported that BA secretion can provide ample energy to support diverse microbiota (48). Such biotransformation can modify the signaling effects of BAs on FXR and TGR5. The gut microbiota may suppress intestinal FXR signaling by altering BA composition, which is related to the regulation of BA homeostasis. Studies have indicated that CYP7A1 is strongly regulated by intestinal FXR (49). When normal mice receive gut microbiota transplants from obese mice, there is an increase in FXR mRNA expression in the ileal tissue, along with an increase in FGF15 mRNA expression, leading to a decrease in hepatic CYP7A1 mRNA expression (50). A comparison of the BA composition in the entire enterohepatic system of germ-free and conventionally raised (CONV-R) mice suggested that the gut microbiota may suppress CYP7A1 and BA synthesis by reducing T-MCA levels and promoting FXR-dependent FGF15 expression in the ileum, indicating that the gut microbiota’s regulation of BAs primarily occurs through the intestinal FXR signaling pathway (51). In this study, SZ-A simultaneously regulated the gut microbiota and BA metabolism while inhibiting ileal FXR-FGF15 signaling, leading to increased expression of hepatic CYP7A1. Consequently, several obesity-related metabolic parameters were improved. Among the BAs, both DCA and LCA are capable of activating FXR expression. Following SZ-A treatment, the levels of DCA and LCA increased, which suppressed the FXR signaling pathway and ultimately contributed to weight reduction.

TGR5, a BA-activated membrane receptor found on the cell surface, exhibits abundant expression in the gallbladder, brown adipose tissue, muscle tissue, and intestinal tract and is pivotal in the regulation of metabolic processes and energy expenditure (52). BAs are potent agonists of TGR5, and TGR5 knockout mice exhibit a 21%–25% decrease in total BA pool levels compared with wild-type mice, highlighting the significant role of TGR5 in maintaining BA balance (53). Certain active components of traditional Chinese medicine, acting as natural TGR5 agonists, can increase BA concentrations and enhance intestinal GLP-1 release, alleviating obesity induced by HFD in mice (54). GLP-1 is an incretin hormone that promotes insulin release, thereby regulating blood glucose levels, gastrointestinal motility, and appetite. As an agonist of TGR5, HDCA may play a role in modulating metabolism and energy expenditure, particularly showing potential therapeutic value in the treatment of metabolic disorders, such as obesity, diabetes, dyslipidemia, atherosclerosis, NAFLD, and neurological conditions (55). Research by Wang et al. has shown that dual agonists of TGR5 and FXR can exert synergistic effects through various pathways, improving the obese state of mice fed an HFD by engaging multiple complementary and nonoverlapping signaling and metabolic pathways (56). A growing body of evidence suggests that BA signaling through FXR and TGR5 can reduce triglycerides and regulate energy metabolism (57), indicating the role of FXR and TGR5 in modulating lipid and energy metabolism in the liver, adipocytes, and intestine.

Leptin, a hormone secreted by adipose tissue, is present in the serum in amounts proportional to the size of an individual’s fat mass (58). In states of obesity, leptin secretion increases. SZ-A modulates leptin levels, inhibiting lipogenesis and promoting lipolysis, thereby regulating lipid metabolism. In the state of obesity, the target cells of insulin can activate inflammatory pathways, leading to insulin resistance. HFD causes rats to become obese and develop dyslipidemia, which in turn leads to increased insulin secretion. SZ-A, on the other hand, reduces BW and improves insulin resistance by regulating gut microbiota and BAs. Overall, insulin, GLP-1, and leptin are involved in the regulation of energy balance and BW; these three hormones are interrelated in the body’s metabolic regulation and work together to maintain homeostasis.

Disruptions in the gut microbiota can lead to overgrowth of pathogenic bacteria, resulting in compromised intestinal barriers and associated low-grade inflammation. Obesity is a low-grade inflammatory disease, and the overexpression of inflammatory cytokines can accelerate its progression. Occludin, claudin-1, and ZO-1 are key proteins that constitute TJs between cells, playing a crucial role in maintaining the barrier functions of epithelial and endothelial cells. These three proteins work in concert to ensure the proper formation and function of cell-to-cell TJs, which is essential for maintaining the integrity of the intestinal barrier, regulating the permeability of paracellular pathways, and preventing the invasion of pathogens and harmful substances. Our study demonstrated that SZ-A mitigated intestinal inflammation induced by HFD by notably escalating the expression of occludin, claudin-1, and ZO-1 and decreasing the levels of the inflammatory cytokines IL-1β, IL-6, and TNF-α in colonic tissue, thereby improving intestinal permeability.

While our research sheds initial light on how SZ-A influences lipid metabolism in HFD-fed rats, it is not without limitations. Future studies should build upon and deepen our understanding of the findings presented here. We observed that SZ-A significantly alters lipid metabolism in these rats, yet these findings do not align perfectly with those from another study (17). The discrepancies may stem from variations in the microbiomes associated with different dietary compositions and animal husbandry conditions across studies, potentially resulting in divergent phenotypic expressions. Through the synthesis of metagenomics, lipidomics, and targeted BA profiling, we identified potential target genes for SZ-A; however, these require further investigation to confirm the annotated discrepancies. Delving into the roles of these pivotal metabolites and their corresponding target genes in the context of obesity and lipid metabolism disorders is set to yield valuable insights. Such explorations will not only enhance our comprehension of SZ-A’s mechanisms of action but may also uncover innovative therapeutic approaches to combat obesity and associated metabolic disorders.

## 5 Conclusion

The gut microbiota plays a pivotal role in modulating BA metabolism, significantly impacting the FXR and TGR5 signaling pathways that are closely associated with BAs. In a bidirectional relationship, BAs also trigger changes in the composition and balance of gut microbiota. Our research has revealed that SZ-A can induce metabolic changes in the host by regulating BA signaling mediated by the gut microbiota, thereby restoring the intestinal barrier and improving inflammatory responses. These effects contribute to the amelioration of obesity and lipid metabolic dysregulation. Consequently, maintaining a balanced gut microbiota and ensuring proper BA metabolism are essential for the prevention and management of obesity and its associated comorbidities.

## Data Availability

The original contributions presented in the study are included in the article/supplementary material. Further inquiries can be directed to the corresponding authors.
